# Factors associated with health-related quality of life in patients with Crohn's disease in Iran: A prospective observational study

**DOI:** 10.3389/fmed.2022.1091330

**Published:** 2023-01-19

**Authors:** Hassan Karami, Maryam Shirvani Shiri, Farbod Ebadi Fard Azar, Kamran Bagheri Lankarani, Sulmaz Ghahramani, Aziz Rezapour, Maryam Tatari, Zahra Heidari Javargi

**Affiliations:** ^1^Department of Economics, School of Health Management and Information Sciences, Iran University of Medical Sciences, Tehran, Iran; ^2^Department of Health Management and Economics, School of Public Health, Tehran University of Medical Sciences, Tehran, Iran; ^3^Health Promotion Research Center, Iran University of Medical Sciences, Tehran, Iran; ^4^Health Policy Research Center, Institute of Health, Shiraz University of Medical Sciences, Shiraz, Fars, Iran; ^5^Health Management and Economics Research Center, Iran University of Medical Sciences, Tehran, Iran; ^6^Department of Epidemiology and Biostatistics, School of Public Health, Tehran University of Medical Sciences, Tehran, Iran; ^7^Vice Chancellery of Health, Torbat Heydariyeh University of Medical Sciences, Torbat Heydariyeh, Iran; ^8^Department of Clinical Medicine, School of Medicine, Tehran Medical Sciences, Islamic Azad University, Tehran, Iran

**Keywords:** health-related quality of life, EQ-5D, IBDQ-9, EQ-VAS, associated factors, Crohn's disease

## Abstract

This was a 1-year prospective observational study of the health-related quality of life (HRQoL) of moderate to severe crohn's disease (CD) patients in Iran. Patients' HRQoL were measured using the EQ-5D 3L, EQ-VAS, and IBDQ-9 tools. HRQoL among CD patients were compared using the *T*-test, Mann–Whitney, Chi-square, and Fisher's exact tests. To discover factors influencing patients' HRQoL, multivariate linear regression and multivariate logistic regression tests were utilized. The study included 222 CD patients, with a mean age of 34.67 and mean disease duration of 7.32 years. The dimensions with the worst reported “relatively or extreme problems” were P/D: 77.5% and A/D: 63.1%. Employment, having “other chronic diseases,” and ADA consumption were the most important independent predictors of HRQoL in CD patients, [β = 0.21 (EQ-5D index), β = 19.61 (EQ-VAS), β = 12.26 (IBDQ-9), OR: 0.09 (MO), OR: 0.12 (UA), OR: 0.21 (P/D), OR: 0.22 (A/D)], [β = −0.15 (EQ-5D index), β = −5.84 (IBDQ-9), β = −11.06 (EQ-VAS), OR: 4.20 (MO), OR: 6.50 (UA)], and [OR: 2.29 (A/D)], respectively. Unemployment, presence of “other chronic conditions” had the greatest negative impact on HRQoL of CD patients. There were significant differences in the probability of reporting “relatively or extreme problems” in the SC and A/D dimensions between patients using adalimumab (ADA) and infliximab (IFX).

## Introduction

CD is an inflammatory disease that can affect any part of the digestive tract, including the mouth, anus, and esophagus. Diarrhea, abdominal pain, anal hemorrhage, and weight loss are among the digestive symptoms of CD, as is skin lesions beyond the gastrointestinal tract. CD is characterized by recovery and recurrence periods (1, 2). According to the World Gastroenterology Organization, CD incidence is 29 out of every 100,000 people in the Middle East, while its incidence is 5 per 100,000 (3).

Surgical admissions, living with a stoma and short bowel syndrome followed by removing an extensive portion of the intestine, and indirect expenses such as lost income, productivity, and leisure time are all linked to HRQoL in CD patients (4).

HRQoL shows a disease's functional influence and is one of the most critical issues in chronic diseases (4). Clinical, psychological, socio-demographic, and treatment-related aspects are all important drivers of HRQoL in CD illness (5, 6). Although disease activity is an important determinant of HRQoL in CD disease, even asymptomatic individuals have lower HRQoL than the general population, demonstrating the importance of other factors in CD patients' HRQoL (7, 8). A detailed grasp of the above-mentioned effects on HRQoL can play a significant role in clinician decisions, particularly when deciding between two therapeutic procedures with the same clinical impact or while treating asymptomatic patients (9). The Crohn's Disease Activity Index (CDAI) is commonly used in clinical trials to quantify the clinical activity of CD disease, but it cannot adequately measure disease burden (10). As a result, evaluating the repercussions of this condition requires more than only monitoring clinical activity. It is also critical to determine patients' HRQoL (11).

According to a study of the literature, the majority of HRQoL research has been conducted on CD patients in western countries. As a result, their findings cannot be applied to CD patients of other nations. Because those countries' health-care structures, as well as their cultural traits and CD-coping mechanisms, differ from other regions (12, 13). Studies have shown that the prevalence and incidence of inflammatory bowel disease (IBD) in Iran, especially in the last two decades has increased significantly (14, 15). According to our best knowledge, no study assessing the HRQoL of CD patients has been undertaken in Iran. As a result, the purpose of this study was to assess the HRQoL in patients with moderate to severe CD who are using IFX or ADA and relationship between socioeconomic variables and patients' HRQoL in Iran. Furthermore, the utility scores of patients' health status can be retrieved and employed in economic evaluation studies utilizing the tool (EQ-5D) used in this study.

## Methods

### Subjects and study design

This prospective and observational study was carried out in Bushehr, Fars, and Yazd provinces between July 2019 and January 2021. The study included severe and moderate CD patients who were treated with IFX or ADA and were followed for a year. The following were the inclusion criteria. (1) Severe and moderate CD patients who were using IFX or ADA at the start of the study (2) Patients who have a file for the specified drugs in their place of residence's Social Security Insurance Office or Iranian Health Insurance Office (these two insurance funds are the largest insurance fund in Iran and cover more than 90% of the population of Iran). In addition, they were diagnosed with severe or moderate CD illness by a clinician using the Harvey-Bradshaw criteria and were prescribed IFX or ADA. According to the Harvey-Bradshaw index, instances with a score between 8 and 16 are classified as moderate CD patients, and cases with a score greater than 16 are classified as severe CD patients. It should be noted that in order to acquire IFX or ADA medication in Iran, CD patients must first produce medical documentation, which includes a report confirming the disease from a gastroenterologist, as well as a prescription to refer to the insurance office and register. 3. Additionally, patients with a valid address and phone number on file at the relevant insurance office and 4. The research comprised patients who were willing and eager to participate in the study. The following are the exclusion criteria: (1) Patients who changed their treatment for any reason throughout the 1-year observation period. (2) Patients who died during their 1-year monitoring period. (3) Patients who were unable to participate in the study due to a variety of factors (lack of access, unwillingness to participate in the study). In this study, stratified random sampling was performed.

### Procedure for collecting the data

After the necessary coordination, complete access to the computerized records of all CD patients became feasible. Then, for all CD patients using IFX and ADA, a list of their characteristics, addresses, and clinical features, such as disease severity, was retrieved, and sampling was performed. After explaining the research objectives and obtaining their consent to participate in the study and ensure the use of ADA and IFX, the patients were contacted. Necessary information such as the type and severity of the disease, address, telephone number, and drugs consumed were recorded. Patients were called again at the ending of the 12-month period, and data collection tools were provided to them. Because of the coronavirus disease (COVID-19) pandemic, this study tool was distributed as an online questionnaire to patients' cellphone numbers *via* WhatsApp, and they were asked to answer to the needed information. For patients who were illiterate or did not have a WhatsApp number, the link to the online questionnaire was supplied to their spouse or children, and following the necessary training, others around the patient were instructed to ask the survey questionnaires of the patient and record the considered answer. The questionnaire had a statement that included the study's objectives as well as informed consent to participate in the study, and patients started the main portion of the questionnaire after verifying these statements. The Iran University of Medical Sciences' ethical committee has approved this study (IR.IUMS.MEDICINE.REC.1398.222).

### Measures

In addition to the patients' electronic records, data was collected using a specially created online questionnaire with three sections.

#### Socio-demographic and clinical history section

Some information was gathered in this part. These data included patients' socio-demographic and clinical history, such as residence, age, marital status, history of smoking and alcohol consumption, education, supplemental insurance, gender, type of disease, history of surgery in the previous year, duration of disease, medication used, employment status, and history of hyperthyroidism, hypertension, diabetes, asthma, malnutrition, and “other chronic diseases”.

#### EuroQol 5-dimensions 3-levels authorized Persian questionnaire

The EuroQol 5-dimensional 3 level (EQ-5D 3L) questionnaire was used to assess patients' health status. The questionnaire is made up of five questions, each measuring one of the five components of HRQoL. Mobility (MO), Self-Care (SC), Usual Activities (UA), Pain/Discomfort (P/D), and Anxiety/Depression (A/D), are among these dimensions. In this study, the Iranian value set retrieved by Goudarzi et al. (16) using the time trade-off (TTO) method was used. EQ-5D scores range from −0.113 (most extremely impairment across all five dimension) to 1 (no problems in any dimension) (16).

The EQ-5D-3L also has an easy-to-use analog scale (EQ-VAS). It assesses an individual's perception of the quality of life on a scale of 0 to 100. The best possible condition of health is one hundred, whereas the poorest possible state of health is zero. This data can be used to calculate the quantitative amount of health outcomes acquired by respondents (17). The Persian versions of the EQ-5D 3L and EQ-VAS have been reported to have acceptable number and percentage (18).

#### Inflammatory Bowel disease questionnaire-short form

It is a unique questionnaire that assesses IBD patients' quality of life in relation to their health. Casellas et al. created the initial edition of the Inflammatory Bowel disease questionnaire-short form (IBDQ-9) questionnaire based on the original version of the IBDQ (19). Through 9 items, this questionnaire assesses patients' quality of life in four dimensions: gastrointestinal, systemic, emotional, and social issues. Each item is graded on a scale of 1 (worst condition) to 7 (best condition). The total score ranges from 9 to 63, with a higher number indicating a better quality of life (20). The Persian version of the IBDQ-9 has been reported to have acceptable validity and reliability (20).

The clinical significance at the stated significance level was evaluated using the minimal clinically important difference (MCID) of the mentioned techniques. The MCID is the smallest number of the difference between the scores that is considered clinically meaningful. And this figure is based on a survey of the literature. The MCID EQ-5D index and EQ-VAS score are 0.08 and 10, respectively (21, 22).

### Statistical evaluation

Quantitative data are expressed as means and standard deviations, while qualitative factors are expressed as number and percentage. The *t*-test was performed to compare the EQ-5D index, EQ-VAS, and IBDQ-9 scores in patients. To compare the ratio of five dimensions of health in patients, the Fisher's exact and Chi-square tests were utilized. Levels two and three of the EQ-5D were integrated and classified as “no problem” or “relatively or extremely problemes” in all five domains of health. To identify determinants of the EQ-5D Index, EQ-VAS, and IBDQ-9 Score, multivariate linear regression was performed. The defaults and requirements of the linear regression approach were examined using normality, linearity, and homoscedasticity, and all hypotheses were established for testing. To enter variables into the model, the Backward Elimination approach was utilized. Multivariate logistic regression was used to calculate odds ratios (ORs) and 95% confidence intervals for determinants of EQ-5D-3L dimensions. All statistical tests were adjusted bilaterally, and the significance threshold was set at 0.05. R3.6.3 software was used for all testing.

## Results

In total, 254 CD patients were contacted and 222 individuals agreed to participate in the study. Table 1 summarizes the demographic and clinical features of the patients. The Mean ± SD age and disease duration of the patients was 34.67 ± 12.01 and 7.318 ± 5.04 years, respectively. According to the results, more than half of the participants were male (53.6%), married (59.9%) and urbanite (73.3%). Also, 35.6% of patients were employed and 49.1% of them had university education. Moreover, 13.9% of patients had a history of surgery in the past year. Of the 222 patients, 83 patients were taking IFX, and 139 patients were taking adalimumab.

**Table 1:** Socio-demographics and clinical characteristics of CD patients.

**Variables**	**Characteristics**	**Mean ±SD/number (percent)**
Number		222
Age (years)		34.67 ± 12.01
Disease duration (years)		7.318 ± 5.04
Gender	Male	119 (53.6)
	Female	103 (46.4)
Marital status	Single/widowed/divorced	89 (40.1)
	Married	133 (59.9)
Employment status	Housewives	58 (26.1)
	Disabled/retired	12 (5.4)
	Unemployed	45 (20.3)
	Student	28 (12.6)
	Employed	79 (35.6)
Residence	Village	59 (26.7)
	Urban	162 (73.3)
Level of education	<6 years	20 (9.0)
	6–12 years	93 (41.9)
	>12 years	109 (49.1)
Supplementary insurance	Yes	90 (40.5)
	No	132 (59.5)
Tobacco use	Yes	32 (14.4)
	No	190 (85.6)
Alcoholic beverages use	Yes	18 (8.1)
	No	204 (91.9)
Surgery in 1 past year	Yes	31 (13.9)
	No	191 (86.1)
Hypertension	Yes	10 (4.5)
	No	212 (95.5)
Diabetes type 2	Yes	8 (3.6)
	No	214 (96.4)
Malnutrition	Yes	8 (3.6)
	No	214 (96.4)
Hypothyroidism	Yes	7 (3.2)
	No	215 (96.8)
Asthma	Yes	2 (0.09)
	No	220 (99.1)
Other chronic disease	Yes	25 (11.3)
	No	197 (88.7)
Main drug	Infliximab	83 (37.39)
	Adalimumab	139 (62.61)

Values are presented as mean ± standard deviation or number (percent).

### EQ-5D dimensions and HRQoL scores of EQ-5D index

According to the MO, SC, UA, P/D, and A/D dimensions, 33.8, 14.9, 36.0, 77.5, and 63.1% of patients, respectively, reported “relatively or extreme problems” (Table 2). The percentage of users reporting “relatively or extreme problems” in the SC dimension was 24.1 in IFX users and 9.4 in ADA users, which were significantly different. The percentages of IFX and ADA users reporting “relatively or extreme problems” in the A/D dimension were 54.2 and 68.3, respectively, which were significantly different (Table 2). The mean and standard deviation of the EQ-5D index were 0.70 and 0.20, respectively, among all patients. This score was 0.56 ± 0.25 in CD patients with “other chronic diseases” and 0.40 ± 0.16 in malnourished CD patients, which was statistically and clinically significantly different from the 0.72 ± 0.19 in CD patients without “other chronic diseases” and 0.71 ± 0.20 in malnourished CD patients, respectively (*P* < 0.05). This measure was 0.69 ± 0.22 in IFX users and 0.71 ± 0.19 in ADA users, but there was no statistically significant difference (Table 3).

**Table 2:** Percentages of patients with “relatively or extreme problems” in each of the five dimensions of EQ-5D stratified by socio-demographic and clinical characteristics.

**Variables**	**Categories**	**Percentage with “relatively or extreme problems” (%)**				
		**Mobility**	**Self-care**	**Usual activity**	**Pain/discomfort**	**Anxiety/** **depression**
Overall		33.8	14.9	36.0	77.5	63.1
Gender	Female	36.9	18.4	38.8	80.6	72.8
	Male	31.1	11.8	33.6	74.8	54.6^*^
Age (years) Mean ± SD		37.7 ± 12.60^*^	37.0 ± 11.8	34.6 ± 10.96	35.3 ± 11.83	34.6 ± 11.11
Disease duration(years) Mean ± SD		7.84 ± 5.41	6.73 ± 5.45	6.89 ± 4.31	7.63 ± 5.21	7.68 ± 5.08
Marital status	Married	38.3	18.0	38.3	82.0	68.4
	Single/widow/divorced	27.0	10.1	32.6	70.8	55.1^*^
Employment status	Housewives	39.7	29.3^*^	39.7	87.9	70.7
	Disabled/retired	41.7	16.7	25.0	75.0	50.0
	Unemployed	51.1^*^	17.8	64.4^*^	86.7	80.0^*^
	Student	7.1	3.6	21.4	57.1	42.9
	Employed	27.8	6.3	24.1	72.2	57.0
Residence	Urban	27.8	13.6	30.9	75.9	64.2
	Village	49.2^*^	18.6	50.8^*^	81.4	59.3
Levels of Education	<6 years	55.0	45.0	40.0	90.0	50.0
	6–12 years	46.2	15.1	49.5	87.1	77.4^*^
	>12 years	19.3^*^	9.2^*^	23.9^*^	67.0^*^	53.2
Supplementary insurance	Yes	31.1	8.9	28.9	74.4	60.0
	No	35.6	18.9^*^	40.9	79.5	65.2
Tobacco use	Yes	28.1	9.4	34.4	71.9	50.0
	No	34.7	15.8	36.3	78.4	65.3
Alcoholic beverages use	Yes	38.9	16.7	27.8	83.3	72.2
	No	33.3	14.7	36.8	77.0	62.3
Surgery in 1 past year	Yes	45.6^*^	19.1	48.5^*^	80.9	57.4
	No	28.6	13.0	30.5	76.0	65.6
Hypertension	Yes	30.0	20.0	40.0	70.0	60.0
	No	34.0	14.6	35.8	77.8	63.2
Diabetes type 2	Yes	50.0	25.0	50.0	100.0	100.0^*^
	No	33.2	14.5	35.5	76.6	61.7
Malnutrition	Yes	87.5^*^	50.0^*^	75.0^*^	87.5	100.0^*^
	No	31.8	13.6	34.6	77.1	61.7
Hypothyroidism	Yes	28.6	14.3	42.9	85.7	71.4
	No	34.0	14.9	35.8	77.2	62.8
Asthma	Yes	50.0	50.0	50.0	50.0	50.0
	No	33.6	14.5	35.9	77.7	63.2
Other chronic disease	Yes	64.0^*^	28.0	72.0^*^	96.0^*^	64.0
	No	29.9	13.2	31.5	75.1	62.9
Drug use	Infliximab	34.9	24.1^*^	41.0	73.5	54.2^*^
	Adalimumab	33.1	9.4	33.1	79.9	68.3

^*^P value < 0.05.

**Table 3:** Mean and standard deviation of EQ-5D index, IBDQ-9 Score and EQ-VAS stratified by socio-demographic and clinical characteristics.

**Variables**	**Categories**	**EQ-5D 3L index (mean ±SD)**	**EQ-VAS (mean ±SD)**	**IBDQ-9 score (mean ±SD)**
Overall		0.70 ± 0.20	62.20 ± 19.87	39.14 ± 11.46
Gender	Female	0.67 ± 0.19	61.09 ± 19.37	39.50 ± 10.91
	Male	0.72 ± 0.20	63.16 ± 20.32	38.83 ± 11.95
Marital status	Married	0.68 ± 0.20	60.05 ± 19.60^*^	38.06 ± 10.41
	Single/widow/divorced	0.73 ± 0.19	65.42 ± 19.94	40.75 ± 12.77
Employment status	Housewives	0.65 ± 0.20	57.59 ± 19.43	37.88 ± 8.44
	Disabled/retired	0.71 ± 0.28	60.75 ± 22.57	40.00 ± 9.66
	Unemployed	0.59 ± 0.19^*^	52.51 ± 20.09^*^	34.58 ± 12.11^*^
	Student	0.82 ± 0.15	74.50 ± 15.97	47.75 ± 9.74
	Employed	0.76 ± 0.16	66.99 ± 17.52	39.48 ± 12.30
Residence	Urban	0.72 ± 0.18	63.77 ± 18.76	40.15 ± 11.32^*^
	Village	0.66 ± 0.24	58.12 ± 22.39	36.68 ± 11.39
Levels of education	<6 years	0.61 ± 0.25	54.15 ± 20.60	38.20 ± 10.35
	6–12 years	0.63 ± 0.18	56.37 ± 19.47	35.55 ± 10.48
	>12 years	0.77 ± 0.17^*^	68.66 ± 18.07^*^	42.38 ± 11.60^*^
Supplementary insurance	Yes	0.73 ± 0.18	63.97 ± 18.90	39.86 ± 11.60
	No	0.68 ± 0.21	61.01 ± 20.49	38.65 ± 11.39
Tobacco use	Yes	0.73 ± 0.21	64.84 ± 21.27	37.75 ± 12.04
	No	0.69 ± 0.19	61.76 ± 19.64	39.37 ± 11.38
Alcoholic beverages use	Yes	0.69 ± 0.16	62.66 ± 19.55	37.44 ± 10.51
	No	0.70 ± 0.20	62.16 ± 19.94	39.29 ± 11.55
Surgery in 1 past year	Yes	0.66 ± 0.22	60.39 ± 20.50	37.32 ± 11.93
	No	0.71 ± 0.19	63.00 ± 19.59	39.94 ± 11.19
Hypertension	Yes	0.72 ± 0.20	63.40 ± 20.57	37.90 ± 11.35
	No	0.70 ± 0.20	62.15 ± 19.88	39.20 ± 11.49
Diabetes type 2	Yes	0.61 ± 0.15^a^	56.25 ± 17.61	35.38 ± 12.55
	No	0.70 ± 0.20	62.43 ± 19.95	39.28 ± 11.43
Malnutrition	Yes	0.49 ± 0.16^*a^	44.75 ± 15.55^*a^	30.63 ± 10.46^*^
	No	0.71 ± 0.20	62.86 ± 19.75	39.46 ± 11.40
Hypothyroidism	Yes	0.72 ± 0.18	65.71 ± 14.84	42.14 ± 8.65
	No	0.70 ± 0.20	62.09 ± 20.03	39.04 ± 11.55
Asthma	Yes	0.73 ± 0.38	65.00 ± 35.36	43.50 ± 17.67
	No	0.70 ± 0.20	62.18 ± 19.82	39.10 ± 11.44
Other chronic disease	Yes	0.56 ± 0.25^*a^	51.44 ± 19.72^*a^	34.04 ± 9.21^*^
	No	0.72 ± 0.19	63.57 ± 19.52	39.79 ± 11.58
Drug use	Infliximab	0.69 ± 0.22	62.49 ± 21.72	38.39 ± 12.23
	Adalimumab	0.71 ± 019	62.04 ± 18.76	39.59 ± 11.00

EQ-5D-3L, EuroQol 5-dimensional 3 level questionnaire; IBDQ-9, inflammatory Bowel disease questionnaire-short form; EQ-VAS, Euro-Qual visual analog scale.

^*^P value < 0.05.

^a^Greater than minimum clinically important difference (MCID).

### HRQoL scores of IBDQ-9

The mean and standard deviation of all patients' IBDQ-9 scores were 39.14 ± 11.46. This score was 34.58 ± 12.11 among unemployed individuals, which was significantly lower than in other patients. Urban inhabitants scored 40.15 ± 11.32, which was much higher than rural dwellers' score of 36.68 ± 11.39. Patients with more than 12 years of education had a substantially higher score (42.38 ± 11.60) than those with less than 12 years of education. Patients who were malnourished had a substantially lower score (30.63 ± 10.46) than those who were not malnourished (39.46 ± 11.40) (*P* < 0.05). IFX using had an index of 38.39 ± 12.23 and ADA using had an index of 39.59 ± 11.00, with no statistically or clinically significant differences (Table 3).

### HRQoL scores of EQ-VAS

The mean and standard deviation of all patients' EQ-VAS scores were 62.20 ± 19.87. This score was 60.05 ± 19.60 among married individuals, which was considerably lower than the 65.42 ± 19.94 in single, widowed, or divorced individuals (*P*-value; 0.04). Unemployed individuals had a substantially lower score of 52.51 ± 20.59, whereas patients with a minimum of 12 years of education had a significantly higher score of 68.66 18.07 (*P* < 0.001). The difference in scores between CD patients without malnutrition (62.86 ± 19.75) and malnourished patients (44.75 ± 15.55), as well as between CD patients with “other chronic diseases” (51.44 ± 19.72) and CD patients without “other chronic diseases” (63.57 ± 19.52), was statistically and clinically significant (*P* < 0.001). Finally, the results indicated that there was no statistically significant difference in the EQ-VAS scores of patients receiving IFX (62.49 ± 21.72) against those receiving ADA (62.04 ± 18.76) (Table 3).

### Factors association with the EQ-5D index

Employees *b* = 0.21, students *b* = 0.15, and persons with “other chronic conditions” *b*= −0.15 all suggested a significant relationship with the EQ-5D Index (*P* < 0.001; Table 4). Other variables exhibited no correlation with the EQ-5D Index.

**Table 4:** Regression coefficients of variables used in multivariate linear regression.

**Variable**	**Categories**	**EQ-5D 3L index**			**IBDQ-9 score**			**EQ-VAS**		

		β	**95% CI**	***P*** **-value**	β	**95% CI**	***P*** **-value**	β	**95% CI**	***P*** **-value**
Employment status	Unemployed	Ref			Ref			Ref		
	Housewives	0.08	0.01,0.14	**0.018**	4.19	0.12,8.24	**0.043**	6.94	0.18,13.70	**0.04**
	Disabled/retired	0.14	0.02,0.24	**0.015**	6.13	−0.47, 12.73	0.069	9.74	−1.25,20.74	0.082
	Employed	0.21	0.12,0.29	**<0.001**	12.26	7.33, 17.18	**<0.001**	19.61	11.41,27.82	**<0.001**
	Student	0.15	0.08,0.21	**<0.001**	4.38	0.56, 8.20	**0.025**	12.83	6.47,19.19	**<0.001**
Other chronic disease	No		Ref			Ref			Ref	
	Yes	−0.15	−0.21, −0.07	**<0.001**	−5.84	−10.20, −1.47	**0.009**	−11.06	−18.34, −3.79	**0.002**

EQ-5D-3L, EuroQol 5-dimensional 3 level questionnaire; IBDQ-9, inflammatory Bowel disease questionnaire-short form; EQ-VAS, Euro-Qual visual analog scale; CI, confidence interval; Ref, reference group; “–”, not shown in the table (multivariate linear regression *P* > 0.05).

Boldness: *P* value < 0.05.

### Factors association with the IBDQ-9

Multivariate linear regression analysis revealed a positive and significant connection between housewives *b* = 4.19 and IBDQ-9. Additionally, employees *b* = 12.26 and students *b* = 4.38 showed a significant positive association with IBDQ-9, but those with “other chronic conditions” *b* = −5.84 had a significant negative link with IBDQ-9 (*P*-value; 0.009; Table 4). Other variables did not have a statistically significant link with the IBDQ-9.

### Factors association with the EQ-VAS

Multivariate linear regression analysis revealed a positive and significant relationship between housewives *b* = 6.94, employees *b* = 19.61, and students *b* = 12.83 and the EQ-VAS (*P* < 0.05). Additionally, individuals with “other chronic conditions” *b* = −11.06 exhibited a statistically significant association with the EQ-VAS (*P*-value; 0.002; Table 4). Other variables did not have a statistically significant relationship with the EQ-VAS.

### Factors association with the EQ-5D dimensions

According to multivariate logistic regression analysis, the probability of reporting “relatively or extreme problems” in the MO dimension was significantly higher in employees (OR = 0.09) and students (OR = 0.50) compared to unemployed people, and in CD patients with “other chronic diseases” (OR = 4.20) compared to CD patients without “other chronic diseases.” There was a significant correlation between the probability of reporting “relatively or extreme problems” in patients with supplementary insurance (OR = 0.41) and ADA users (OR = 0.19) compared to patients without supplementary insurance and IFX users in the SC dimension. There was a significant difference in the probability of reporting “relatively or extreme problems” in the UA dimension between housewives (OR = 0.28), retirees/disabled (OR = 0.13), employees (OR = 0.12), and students (OR = 0.17) compared to the unemployed, and CD patients with “other chronic diseases” (OR = 6.50) compared to CD patients without “other chronic diseases.” Additionally, there was a significant relationship between the probability of reporting “relatively or extreme problems” among employees (OR = 0.21) and students (OR = 0.41) when compared to unemployed people in the P/D dimension. Finally, there was a significant difference in the likelihood of reporting “relatively or extreme problems” in the P/D dimension between housewives (OR = 0.30), retirees/disabled (OR = 0.22), and students (OR = 0.43) compared to the unemployed, alcoholics (OR = 5.86) compared to non-alcoholics, smokers (OR = 0.5) compared to non-smokers, and ADA users (OR = 2.29) compared to IFX users (*P* < 0.05; Table 5). Other variables exhibited no correlation with the EQ-5D dimensions.

**Table 5:** Variables associated with “relatively or extreme problems” in each EQ-5D 3L dimensions using multivariate logistic regression.

**Variable**	**Categories**	**Odds ratio (95% CI)**				

		**Mobility**	**Self-care**	**Usual activity**	**Pain/discomfort**	**Anxiety/** **depression**
Employment status	Unemployed	1.0 (ref.)	–	1.0 (ref.)	1.0 (ref.)	1.0 (ref.)
	Housewives	–	–	0.28 (0.12, 0.66)	–	–
	Disabled /retired	–	–	0.13 (0.02, 0.64)	–	0.30 (0.05, 0.98)
	Employed	0.09 (0.02,0.41)	–	0.12 (0.04, 0.39)	0.21 (0.08, 0.53)	0.22 (0.04, 0.39)
	Student	0.50 (0.26,0.96)	–	0.17 (0.07, 0.38)	0.41 (0.20, 0.84)	0.43 (0.10, 0.62)
Supplementary insurance	No	–	1.0 (ref.)	–	–	–
	Yes	–	0.41 (0.11, 0.81)	–	–	–
Alcoholic beverages use	No	–	–	–	–	1.0 (ref.)
	Yes	–	–	–	–	5.86 (1.56, 22.05)
Tobacco use	No	–	–	–	–	1.0 (ref.)
	Yes	–	–	–	–	0.5 (0.11,0.71)
Other chronic disease	No	1.0 (ref.)	–	1.0 (ref.)	–	–
	Yes	4.20 (1.64, 10.71)	–	6.50 (2.45, 17.2)	–	–
Drug use	Infliximab	–	1.0 (ref.)	–	–	1.0 (ref.)
	Adalimumab	–	0.19 (0.08, 0.42)	–	–	2.29 (1.51, 5.59)

CI, confidence interval; Ref, reference group; “–”, not shown in the table (multivariate logistic regression *P* > 0.05). Data shown in table, *P* < 0.05.

## Discussion

The purpose of this study was to assess the HRQoL of CD patients, as well as the factors influencing their HRQoL using general tools EQ-5D 3L and EQ-VAS, as well as the specific IBDQ-9. We used EQ-5D-3L for two reasons, first using the EQ-5D-3L instrument able us to convert the utility scores into QALY for use in economic evaluations of new medications. Second the shorter completion time in contrast to other generic instruments. Moreover, the IBDQ-9 is the most common tool for measuring HRQoL in IBD patients. It is a responsive questionnaire and brisk reflection of HRQoL changes. This is, to the best of our knowledge, the first time this study has been undertaken in Iran. According to the findings of our study, the mean EQ-5D Index of all CD patients was 0.70 ± 0.20. According to a survey of the literature, the mean score of this test in CD patients in Spain was 0.76 ± 0.18 (23), and the median (interquartile range) score of this measure in another study (24) was 0.816 (0.754–0.914). Differences in the value set produced by each country (due to differences in the type of calculation method and demographic characteristics), as well as differences in age and gender composition, level of education, factors related to health care systems, income, and study design, may explain differences in EQ-5D Index scores across studies. In Iran, the mean EQ-5D index for type 2 diabetes was 0.64 (SD, 0.27) (25), thalassemia major was 0.86 (95% CI: 0.83–0.89) (26), ulcerative colitis 0.67 (SD, 0.16) (27), psoriasis was 0.62 (SD, 0.37) (28), and colorectal cancer was 0.45 (SD, 0.03) (29). All CD patients had an average EQ-VAS score of 62.20 (SD, 19.87). Other studies found mean scores of this instrument of 66.8 (SD, 19.2) and median (interquartile range) 60.5 (50–85) (24, 25). Many factors, including cultural, economic, and social differences, might influence a person's perception and feeling in a given scenario. Our findings also revealed that the mean EQ-5D Index and EQ-VAS score of CD patients in this study was significantly lower than the mean score of Iran's general population (Supplementary Table 1). In other countries, the results were similar (30–32). In our study, the average IBDQ-9 score of all patients was 39.14 (SD, 11.46). In one study, the median (interquartile range) score of this instrument in IBD patients receiving standard care was 38.50 (33.25–46.75), in standard care with in-person visits technique was 42.00 (33.75–47.50), and in nurse-assisted telephone care method was 37.50 (28.75–46.25) (25).

According to the findings of our study, the mean EQ-5D Index of patients using IFX and ADA was 0.69 and 0.71, respectively, with no statistically or clinically significant differences between them. The EQ-VAS and IBD-9 instruments were also used in this study, however we found no statistically or clinically significant differences between patients on ADA and IFX. However, there was a significant difference between the two groups in two of the five EQ-5D health dimensions. The percentages of reported “relatively or extreme problems” in the SC dimension when taking IFX and ADA were 24.1% and 9.4%, respectively, and 54.2% and 68.3% in the A/D dimension, which were substantially different in both dimensions. A study of the literature revealed that prior trials evaluating the efficacy of ADA and IFX in Crohn's patients mainly assessed the medications' clinical efficacy (33–38). Other studies have investigated the efficacy of biological medications to non-biologic drugs in people with Crohn's disease or IBD (39, 40).

Using multivariate linear regression analysis, we explored the factors influencing HRQoL in CD patients. Our findings revealed that job factors and “having chronic conditions” had a substantial impact on HRQoL. When compared to unemployed individuals, employed patients had the best HRQoL status. Thus, they had 0.21, 19.61, and 12.26% higher mean scores than unemployed patients on the EQ-5D Index, EQ-VAS, and IBDQ-9, respectively. Several studies have validated our findings (41–45). However, Habibi et al. discovered no statistically significant link between IBDQ-32 score and job status (46). Most people's life revolves around their jobs. Individuals' financial (income stability, social safety) and social (self-esteem and identity, social contacts) requirements are met by the job. People with higher incomes have fewer concerns about these items and, as a result, are likely to have higher utility levels. We also discovered that CD patients who also had “other chronic conditions” had a lower quality of life than CD patients who did not have “other chronic diseases”. As a result, patients with the EQ-5D Index (−0.15%), IBDQ-9 (−5.84%), and EQ-VAS (−11.06%) had lower mean scores than patients without additional chronic conditions. Pizzi et al. (47) also demonstrated that chronic disorders, particularly cardiovascular disease and arthritis, had the most negative influence on HRQoL in IBD patients. Monitoring and treating other chronic conditions in CD patients can have a major impact on HRQoL promotion. As a result, in addition to monitoring and managing CD, health authorities must devise and implement particular operational plans to manage and monitor other chronic diseases that affect CD patients.

Using multivariate logistic regression analysis, we investigated the factors influencing each of the EQ-5D health dimensions. The findings revealed that employment and having “other chronic conditions” had a significant effect on four (MO, UA, P/D, and P/D) and two (MO/UA) of the EQ-5D health dimensions, respectively. Our findings revealed that many variables had a substantial effect on the A/D dimension. We discovered that the likelihood of reporting “relatively or extreme problems” in the A/D dimension is much lower in employed patients than in other individuals. Previous research has confirmed our findings (48, 49). We found that patients who consumed alcohol were five times more likely than those who did not to report “relatively or extreme problems”with the A/D dimension. Other studies agreed with us (50). Our findings revealed that smokers were less likely than non-smokers to report A/D issues. While Magalhães et al. (39) found no significant link between smoking and HRQoL in IBD patients, Severs et al. (51) found a substantial negative link between smoking and HRQoL. More research is needed to validate or refute prior findings. Finally, our study found that patients using ADA were 2.29 times more likely than patients taking IFX to report “relatively or extreme problems” in the A/D dimension. Additional research is needed to develop more proof of the influence of these medications on various aspects of patients' health, particularly the A/D component.

There are several strengths and limitations of our study that merit consideration in interpreting results. This study was the first to evaluate the effectiveness of biologic drugs (IFX and ADA) in the treatment of DC in Iran. At the same time, we used general and specific tools to measure patients' HRQoL. All study participants were patients who did not respond to conventional treatments (anti-inflammatory drugs, steroid medications and antibiotics) and had received one of the biological drugs (ADA or IFX) according to the specialist physician's prescription. Participants in this research included patients who had previously been exposed to biological drugs or patients who were using biological medications for the first time. Therefore, bias may have arisen in the conclusion of our research. We only evaluated the HRQoL of the patients who had used IFX and ADA for 1 year, and we did not evaluate the health outcomes of those who changed their drug during the year due to nonresponse to the medication, as well as those who were excluded from the study for other reasons.

## Conclusion

This study gives EQ-5D index score estimates that can be used in health economics evaluations. The A/D and P/D dimensions are where we see the greatest reports of “relatively or extreme problems”. Psychological counseling programs, in addition to medical measures, appear to be necessary for improving patients' health. Patients who were unemployed, malnourished, or had “other chronic conditions” had much higher “relatively or extreme problems” in most dimensions of health, whereas patients who had a university education had significantly less “relatively or extreme problems” in most dimensions of health. Even after adjusting for all other variables, unemployed patients and patients with “other chronic conditions” in patients with moderate to severe CD on use of IFX or ADA had lower HRQoL than other patients. As a result, we believe that health care practitioners should be aware of not only their patients' clinical parameters, but also of their patients' “other chronic diseases,” job situation and nutritional behavior. Finally, we found no statistically significant difference in the EQ-5D Index, EQ-VAS, and IBDQ-9 scores between patients using ADA and IFX. However, there were a statistically significant difference in the SC and A/D dimensions between patients using ADA and IFX. We highly advise undertaking a cost-effectiveness study from the standpoint of the Iranian community, in which both the outcomes and expenses are examined concurrently.

## Data availability statement

The raw data supporting the conclusions of this article will be made available by the authors, without undue reservation.

## Ethics statement

The studies involving human participants were reviewed and approved by Iran University of Medical Sciences Ethical Committee. The patients/participants provided their written informed consent to participate in this study.

## Author contributions

HK, FE, KB, AR, SG, and MS conceived the experiments. HK and MS performed the experiments. MT and HK analyzed the data. All authors contributed to the article and approved the submitted version.

## References

[1] Tomazoni EI Benvegnú DM. Symptoms of anxiety and depression, and quality of life of patients with Crohn's disease. Arq Gastroenterol. (2018) 55:148–53. 10.1590/s0004-2803.201800000-26 30043864

[2] Mekhjian HS Switz DM Melnyk CS Rankin GB Brooks RK. Clinical features and natural history of Crohn's disease. Gastroenterology. (1979) 77(4, Part 2):898–906. 10.1016/0016-5085(79)90389-5 381094

[3] Bernstein CN Eliakim A Fedail S Fried M Gearry R Goh K-L. World gastroenterology organisation global guidelines inflammatory bowel disease: update august 2015. J Clin Gastroenterol. (2016) 50:803–18. 10.1097/MCG.0000000000000660 27741097

[4] Stanic Benic M Giljaca V Vlahovic-Palcevski V. The impact of biological interventions on health-related quality of life in adults with Crohn's disease. Cochrane Database Syst Rev. (2018) 2018:CD012973. 10.1002/14651858.CD012973 PMC12270664 40678697

[5] Cohen RD. The quality of life in patients with Crohn's disease. Aliment Pharmacol Ther. (2002) 16:1603–9. 10.1046/j.1365-2036.2002.01323.x 12197839

[6] Sainsbury A Heatley RV. Review article: psychosocial factors in the quality of life of patients with inflammatory bowel disease. Aliment Pharmacol Ther. (2005) 21:499–508. 10.1111/j.1365-2036.2005.02380.x 15740531

[7] Mussell M Böcker U Nagel N Singer MV. Predictors of disease-related concerns and other aspects of health-related quality of life in outpatients with inflammatory bowel disease. Eur J Gastroenterol Hepatol. (2004) 16:1273–80. 10.1097/00042737-200412000-00007 15618832

[8] Graff LA Walker JR Lix L Clara I Rawsthorne P Rogala L. The relationship of inflammatory bowel disease type and activity to psychological functioning and quality of life. Clin Gastroenterol Hepatol. (2006) 4:1491–501.e1. 10.1016/j.cgh.2006.09.027 17162241

[9] Van der have M Van der Aalst KS Kaptein AA Leenders M Siersema PD Oldenburg B. Determinants of health-related quality of life in Crohn's disease: a systematic review and meta-analysis. J Crohns Colitis. (2014) 8:93–106. 10.1016/j.crohns.2013.04.007 23746864

[10] Loftus EV Feagan BG Colombel J-F Rubin DT Wu EQ Yu AP. Effects of adalimumab maintenance therapy on health-related quality of life of patients with Crohn's disease: patient-reported outcomes of the charm trial. Am J Gastroenterol. (2008) 103:3132–41. 10.1111/j.1572-0241.2008.02175.x 18853973

[11] Casellas F Robles V Borruel N Torrejón A Castells I Navarro E. Restoration of quality of life of patients with inflammatory bowel disease after one year with antitnfα treatment. J Crohns Colitis. (2012) 6:881–6. 10.1016/j.crohns.2012.01.019 22398074

[12] Levenstein S Li Z Almer S Barbosa A Marquis P Moser G. Cross-cultural variation in disease-related concerns among patients with inflammatory bowel disease. Am J Gastroenterol. (2001) 96:1822–30. 10.1111/j.1572-0241.2001.03878.x 11419836

[13] Petrak JH Clement T Börner N Egle UT Hoffmann SO. Impaired health-related quality of life in inflammatory bowel diseases psychosocial impact and coping styles in a national German sample. Scand J Gastroenterol. (2001) 36:375–82. 10.1080/003655201300051171 11336162

[14] Taghavi SA Safarpour AR Hosseini SV Noroozi H Safarpour M Rahimikazerooni S. Epidemiology of inflammatory bowel diseases (IBD) in Iran: a review of 740 patients in Fars province, southern Iran. Iran J Colorectal Res. (2013) 1:1–2. 10.5812/acr.11477

[15] Safarpour AR Hosseini SV Mehrabani D. Epidemiology of inflammatory bowel diseases in Iran and Asia; a mini review. Iran J Med Sci. (2013) 38:140–9. 24031103 PMC3771215

[16] Goudarzi R Sari AA Zeraati H Rashidian A Mohammad K Amini S. Valuation of quality weights for euroqol 5-dimensional health states with the time trade-off method in the capital of Iran. Value Health Reg Issues. (2019) 18:170–5. 10.1016/j.vhri.2019.01.007 31096140

[17] Euroqol - a new facility for the measurement of health-related quality of life. Health Policy. (1990) 16:199–208. 10.1016/0168-8510(90)90421-9 10109801

[18] Zare F Ameri H Madadizadeh F Aghaei MR. Validity and reliability of the eq-5d-3l (a generic preference-based instrument used for calculating quality-adjusted life -years) for patients with type 2 diabetes in Iran. Diabetes Metab Syndr. (2021) 15:319–24. 10.1016/j.dsx.2021.01.009 33486224

[19] Casellas F Alcalá M-J Prieto L Miró J-RA Malagelada J-R. Assessment of the influence of disease activity on the quality of life of patients with inflammatory bowel disease using a short questionnaire. Am J Gastroenterol. (2004) 99:457–61. 10.1111/j.1572-0241.2004.04071.x 15056085

[20] Gholamrezaei A Haghdani S Shemshaki H Tavakoli H Emami MH. Linguistic validation of the inflammatory bowel disease questionnaire-short form (IBDQ-9) in Iranian population. J Isfahan Med Sch. (2011) 28:1–10.

[21] Coteur G Feagan B Keininger DL Kosinski M. Evaluation of the meaningfulness of health-related quality of life improvements as assessed by the sf-36 and the eq-5d vas in patients with active Crohn's disease. Aliment Pharmacol Ther. (2009) 29:1032–41. 10.1111/j.1365-2036.2009.03966.x 19222413

[22] Stark RG Reitmeir P Leidl R König H-H. Validity, reliability, and responsiveness of the eq-5d in inflammatory bowel disease in Germany. Inflamm Bowel Dis. (2009) 16:42–51. 10.1002/ibd.20989 19475674

[23] Huamán J Casellas F Borruel N Peláez A Torrejón A Castells I. Cutoff values of the inflammatory bowel disease questionnaire to predict a normal health related quality of life? J Crohns Colitis. (2010) 4:637–41. 10.1016/j.crohns.2010.07.006 21122573

[24] Del Hoyo J Nos P Faubel R Muñoz D Domínguez D Bastida G. A web-based telemanagement system for improving disease activity and quality of life in patients with complex inflammatory bowel disease: pilot randomized controlled trial. J Med Internet Res. (2018) 20:e11602. 10.2196/11602 30482739 PMC6301812

[25] Karami H Shirvani Shiri M Rezapour A Sarvari Mehrabadi R Afshari S. The association between diabetic complications and health-related quality of life in patients with type 2 diabetes: a cross-sectional study from Iran. Qual Life Res. (2021) 30:1963–74. 10.1007/s11136-021-02792-7 33900519

[26] Javanbakht M Keshtkaran A Shabaninejad H Karami H Zakerinia M Delavari S. Comparison of blood transfusion plus chelation therapy and bone marrow transplantation in patients with β-thalassemia: application of sf-36, eq-5d, and visual analogue scale measures. Int J Health Policy Manag. (2015) 4:733–40. 10.15171/ijhpm.2015.113 26673333 PMC4629698

[27] Karami H Ebadi Fard Azar F Lankarani KB Rezapour A Ghahramani S Baghbanian A. Adalimumab versus infliximab treatment outcome in ulcerative colitis: application of eq-5d, visual analogue scale, and ibdq-9 measures: a prospective observational study. Curr Drug Saf. (2022). 36029076 10.2174/1574886317666220526153518

[28] Moradi M Rencz F Brodszky V Moradi A Balogh O Gulácsi L. Health status and quality of life in patients with psoriasis: an Iranian cross-sectional survey. Arch Iran Med. (2015) 18:153–9. 25773688

[29] Atfannezhad M Sharifi M Madadizadeh F Ameri H. Utility values in colorectal cancer patients treated with chemotherapy. Cancer Invest. (2022) 40:46–54. 10.1080/07357907.2021.1992632 34634994

[30] Bernklev T Jahnsen J Lygren I Henriksen M Vatn M Moum B. Health-related quality of life in patients with inflammatory bowel disease measured with the short form-36: psychometric assessments and a comparison with general population norms. Inflamm Bowel Dis. (2005) 11:909–18. 10.1097/01.mib.0000179467.01748.99 16189421

[31] Nordin K Påhlman L Larsson K Sundberg-Hjelm M Lööf L. Health-related quality of life and psychological distress in a population-based sample of swedish patients with inflammatory bowel disease. Scand J Gastroenterol. (2002) 37:450–7. 10.1080/003655202317316097 11989837

[32] Schirbel A Reichert A Roll S Baumgart DC Büning C Wittig B. Impact of pain on health-related quality of life in patients with inflammatory bowel disease. World j Gastroenterol. (2010) 16:3168–77. 10.3748/wjg.v16.i25.3168 20593502 PMC2896754

[33] Benmassaoud A Al-Taweel T Sasson MS Moza D Strohl M Kopylov U. Comparative effectiveness of infliximab versus adalimumab in patients with biologic-naïve Crohn's disease. Dig Dis Sci. (2018) 63:1302–10. 10.1007/s10620-017-4874-6 29243105

[34] Varma P Paul E Huang C Headon B Sparrow MP A. retrospective comparison of infliximab versus adalimumab as induction and maintenance therapy for Crohn disease. Intern Med J. (2016) 46:798–804. 10.1111/imj.13040 26865349

[35] Narula N Kainz S Petritsch W Haas T Feichtenschlager T Novacek G. The efficacy and safety of either infliximab or adalimumab in 362 patients with anti-TNF-α naïve Crohn's disease. Aliment Pharmacol Ther. (2016) 44:170–80. 10.1111/apt.13671 27226407

[36] Kestens C Van oijen MGH Mulder CLJ Van bodegraven AA Dijkstra G De jong D. Adalimumab and infliximab are equally effective for Crohn's disease in patients not previously treated with anti–tumor necrosis factor-α agents. Clin Gastroenterol Hepatol. (2013) 11:826–31. 10.1016/j.cgh.2013.01.012 23376000

[37] Di domenicantonio R Trotta F Cascini S Agabiti N Kohn A Gasbarrini A. Population-based cohort study on comparative effectiveness and safety of biologics in inflammatory bowel disease. Clin Epidemiol. (2018) 10:203–13. 10.2147/CLEP.S150030 29440933 PMC5804123

[38] Tursi A Elisei W Picchio M Penna A Lecca PG Forti G. Effectiveness and safety of infliximab and adalimumab for ambulatory Crohn's disease patients in primary gastroenterology centres. Eur J Intern Med. (2014) 25:485–90. 10.1016/j.ejim.2014.02.010 24631020

[39] Magalhães J. Castro FDd, Carvalho PB, Moreira MJ, Cotter J. Quality of life in patients with inflammatory bowel disease: importance of clinical, demographic and psychosocial factors. Arq de Gastroenterol. (2014) 51:192–7. 10.1590/S0004-28032014000300005 25296078

[40] AlRuthia Y Almadi M Aljebreen A Azzam N Alsharif W Alrasheed H. The cost-effectiveness of biologic versus non-biologic treatments and the health-related quality of life among a sample of patients with inflammatory bowel disease in a tertiary care center in Saudi Arabia. J Med Econ. (2020) 23:1102–10. 10.1080/13696998.2020.1791889 32619388

[41] Høivik ML Bernklev T Solberg IC Cvancarova M Lygren I Jahnsen J. patients with Crohn's disease experience reduced general health and vitality in the chronic stage: ten-year results from the Ibsen study?. J Crohns Colitis. (2012) 6:441–53. 10.1016/j.crohns.2011.10.001 22398064

[42] Williet N Sarter H Gower-rousseau C Adrianjafy C Olympie A Buisson A. Patient-reported outcomes in a French nationwide survey of inflammatory bowel disease patients. J Crohns Colitis. (2016) 11:165–74. 10.1093/ecco-jcc/jjw145 27516406

[43] Huppertz-hauss G Lie høivik M Jelsness-jørgensen L-P Henriksen M Høie O Jahnsen J. Health-related quality of life in patients with inflammatory bowel disease 20 years after diagnosis: results from the Ibsen study. Inflamm Bowel Dis. (2016) 22:1679–87. 10.1097/MIB.0000000000000806 27206016

[44] Slonim-nevo V Sarid O Friger M Schwartz D Chernin E Shahar I. Effect of psychosocial stressors on patients with Crohn's disease: threatening life experiences and family relations. Eur J Gastroenterol Hepatol. (2016) 28:1073–81. 10.1097/MEG.0000000000000666 27203602

[45] Parra RS Chebli JMF Amarante HMBS Flores C Parente JML Ramos O. Quality of life, work productivity impairment and healthcare resources in inflammatory bowel diseases in Brazil. World J Gastroenterol. (2019) 25:5862–82. 10.3748/wjg.v25.i38.5862 31636478 PMC6801193

[46] Habibi F Habibi ME Gharavinia A Mahdavi SB Akbarpour MJ Baghaei A. quality of life in inflammatory bowel disease patients: a cross-sectional study. J Res Med Sci. (2017) 22:104. 10.4103/jrms.JRMS_975_16 29026420 PMC5629832

[47] Pizzi LT Weston CM Goldfarb NI Moretti D Cobb N Howell JB. Impact of chronic conditions on quality of life in patients with inflammatory bowel disease. Inflamm Bowel Dis. (2006) 12:47–52. 10.1097/01.MIB.0000191670.04605.e7 16374258

[48] De Boer AGEM Bennebroek Evertsz' F Stokkers PC Bockting CL Sanderman R Hommes DW. Employment status, difficulties at work and quality of life in inflammatory bowel disease patients. Eur J Gastroenterol Hepatol. (2016) 28:1130–6. 10.1097/MEG.0000000000000685 27340897

[49] Bernklev T Jahnsen J Henriksen M Lygren I Aadland E Sauar J. Relationship between sick leave, unemployment, disability, and health-related quality of life in patients with inflammatory bowel disease. Inflamm Bowel Dis. (2006) 12:402–12. 10.1097/01.MIB.0000218762.61217.4a 16670530

[50] Kamp KJ Stommel M. Health-related quality of life among patients with inflammatory bowel disease: a case control study. Gastroenterol Nurs. (2021) 44:21–30. 10.1097/SGA.0000000000000491 33351519

[51] Severs M Mangen M-JJ Van der valk ME Fidder HH Dijkstra G Van der have M. Smoking is associated with higher disease-related costs and lower health-related quality of life in inflammatory bowel disease. J Crohns Colitis. (2016) 11:342–52. 10.1093/ecco-jcc/jjw160 27647859

