# Supplementary material for: Factors associated with health-related quality of life in patients with Crohn's disease in Iran: A prospective observational study
Source: Front Med (Lausanne). 2023 Jan 19;9:1091330. doi: 10.3389/fmed.2022.1091330 (PMC9907090; doi:10.3389/fmed.2022.1091330)
Supplement: Supplementary file 1 [file Table_1.pdf]

Supplementary Table 1. EQ-5D 3L Index and EQ-VAS scores of Study Population and Iranian's General Population

	CD patients (n=222)	Iranian population norms (n=998)	Mean difference	P-value	Reference
	Mean(SD)	Mean(SD)			
EQ-5D 3L Index	0.7(0.20)	0.74(0.19)	-0.04	**0.003**	**[1]**
EQ-VAS	62.2(19.87)	80.9(16.5)	-18.7	**<0.001**	**[1]**
	N (%) any problem	N (%) any problem	-		
Mobility	75(33.8%)	128(12.8)	-	**<0.001**	**[1]**
Self-Care	33(14.9)	89(8.9)	-	**<0.001**	**[1]**
Usual Activity	80(36)	190(19)	-	**<0.001**	**[1]**
Pain/Discomfort	172(77.5)	328(32.9)	-	**<0.001**	**[1]**
Anxiety/Depression	140(63.1)	352(35.3)	-	**<0.001**	**[1]**

EQ-5D-3L, EuroQol 5 Dimensional 3 Level questionnaire; EQ-VAS, Euro-Qual visual analog scale; SD, Standard Deviation; Boldness: P value <0.05.

1. Rezaei S, Hajizadeh M, Kazemi A, Khosravipour M, Khosravi F, Rezaeian S. Determinants of health-related quality of life in Iranian adults: evidence from a cross-sectional study. Epidemiol Health. 2017;39:e2017038-e.[DOI: 10.4178/epih.e2017038]
